# Treatment of Toddler’s Fractures: Study protocol for a multicentre non-inferiority RCT of no-immobilisation or immobilisation

**DOI:** 10.1371/journal.pone.0354801

**Published:** 2026-08-03

**Authors:** Katie Ridsdale, Shammi Ramlakhan, Ines Rombach, Chris Turtle, Steve Goodacre, Daniel C. Perry, Lizzie Swaby, Joshua Milner, Anju Keetharuth, Muniba Aslam, Richard Napier, Robin Chatters, Fay Benskin, Sheryl Bennett, Nicolas Nicolaou, on behalf of the ToTs Study Group

**Affiliations:** 1 School of Medicine and Population Health, University of Sheffield, Sheffield, United Kingdom; 2 Sheffield Children’s Hospital, Sheffield, United Kingdom; 3 University of Liverpool, Liverpool, United Kingdom; 4 Belfast Health and Social Care Trust, Belfast, United Kingdom; 5 Public Representative, Sheffield, United Kingdom; PLOS: Public Library of Science, UNITED KINGDOM OF GREAT BRITAIN AND NORTHERN IRELAND

## Abstract

**Aims:**

Toddler’s fractures are non-displaced spiral fractures of the lower third of the tibia which are usually sustained during a minor fall. These fractures are surrounded by a thick outer lining of bone (the periosteum), which typically makes them stable. Treatment approaches vary; most clinicians use casts or boots to immobilise the leg, whereas others observe recovery without immobilisation. Both approaches are thought to be safe. Whilst immobilisation protects the leg, it can cause issues with stiffness, recovery and complications. The aim of the ToTs study is to determine whether no-immobilisation is non-inferior to immobilisation in young children with toddler’s fractures.

**Methods:**

This randomised controlled trial aims to recruit 494 participants aged 9 months up to their 4th birthday, who present with clinically suspected or radiologically confirmed toddler’s fractures. Most participants are expected to be recruited from emergency departments or fracture clinics. Patients will be randomly allocated 1:1, to either immobilisation or no-immobilisation. The primary outcome is the FLACC (Face, Legs, Activity, Cry, Consolability) behavioural pain assessment completed by parents/guardians at 7 days post randomisation. The primary comparison will assess non-inferiority using a margin of 1 point on the FLACC scale. Secondary outcomes include pain at 3 and 28 days, hospital attendances, need for treatment of pressure ulcers and fracture displacement after randomisation, recovery of mobility, analgesic use, resource use, and satisfaction, collected either by review of medical notes at 28 days, or parent/guardian questionnaires. The cost-effectiveness of the different treatment strategies will also be assessed. Patient and Public Involvement and Engagement informed the study design and will assist with aspects of trial delivery and dissemination.

**Discussion:**

The ToTs trial addresses a lack of high-level evidence regarding the management of toddler’s fractures. This will generate evidence to guide clinical practice regarding whether these fractures should be immobilised.

## Introduction

### Background

Toddler’s fractures are non-displaced spiral fractures of the tibia that affect up to 0.25% of children [1], often after a twisting injury to the leg following a minor stumble or fall. These injuries occur in the lower third of the tibia, and due to the thick periosteal lining, they are stable and heal in a matter of weeks without displacement or long-term problems. Children typically present with an inability to bear weight normally through the leg. A fracture is sometimes apparent on the initial x-ray but does not need to be visible for a toddler’s fracture to be diagnosed.

Treatment approaches vary significantly [2,3]. Many clinicians use casts or boots to immobilise the limb for a varying amount of time. However, others observe recovery without any immobilisation, with pain relief as required.

There are advantages and disadvantages of both treatment methods. Immobilisation, either by cast or boot, allows the limb to be held still, relieving potential discomfort from the fracture associated with joint movement and can maintain the ability to weight bear while the fracture heals. However, it can be associated with other complications including pressure ulcers, skin breakdown, stiffness of the ankle and knee joint, as well as pain from the cast rubbing [1]. It also can lead to delayed recovery from the injury by limiting movement and causing temporary stiffness and weakness of the limb. Beyond clinical issues, it can also impact on daily life activities for children and their families, with casts causing more interference than boots [4].

A conservative approach (no-immobilisation) provides less peace of mind to parents and clinicians. However, it offers potential benefits of a quicker recovery from the injury as it prevents stiffness developing, avoids the risk of complications from the cast or boot, and requires less hospital visits. Since toddler’s fractures are inherently stable injuries, they are not expected to displace if not immobilised. Furthermore, if a diagnosis is uncertain, avoiding immobilisation prevents delays in identifying other important conditions such as bone or joint infections.

A systematic review which included 963 participants (722 immobilised) from 10 studies found no significant difference in discomfort, pain, or fracture-related adverse outcomes between immobilised and non-immobilised children, but 14.7% of immobilised children experienced non-fracture related adverse events (pressure sores, fitting issues, breakage, pain, skin-related issues) [1]. A more recent RCT found higher complication rates in the immobilised group and higher satisfaction in the non-immobilised group. However, the study had several limitations, including a small sample size, the use of both randomisation and treatment preference, differences in treatment preferences by race, and no follow-up during the first four weeks, when pain is likely to be greatest [5].

### Rationale

While both immobilisation and no-immobilisation are currently accepted as standard of care, there is wide regional variation in management. Immobilisation remains the more prevalent approach, driven by an assumption from both clinicians and parents/guardians that stabilisation is necessary for pain control and to prevent displacement.

Despite this, there is a lack of high-quality research into which treatment approach is optimal. There is therefore a requirement for a definitive, robust randomised controlled trial comparing the approaches. No-immobilisation may offer advantages in terms of the avoidance of immobilisation-related risks, convenience for families, and cost-effectiveness. Early Patient and Public Involvement and Engagement (PPIE) work revealed that while families expressed a preference for immobilisation due to perceiving it as safer, they would be happy to forgo it if evidence showed it did not improve pain or reduce complications.

A non-inferiority trial design will be employed to determine whether these advantages can be achieved without an unacceptable increase in pain experienced, as well as provide more insight into parent/guardian satisfaction, recovery, adverse events, and costs. This will provide the evidence needed to standardise practice.

### Aims and objectives

The aim of this study is to evaluate whether no-immobilisation is a safe alternative to immobilisation for the management of suspected or radiologically confirmed toddler’s fractures in children aged from 9 months up to and no later than their 4th birthday.

Objectives:

1. Primary: To determine whether no-immobilisation is non-inferior to immobilisation regarding pain at 7 days.
2. To compare patients’ and parent/guardians’ satisfaction, patient recovery and complications between the two treatments.
3. To evaluate the relative cost-effectiveness by undertaking a within-trial economic evaluation from both a National Health Service (NHS) and a societal perspective.

## Methods

### Trial design

ToTs is a multicentre, prospective, parallel group, individually randomised (1:1), pragmatic, non-blinded, controlled non-inferiority trial with 28 day follow up and within-trial health economic analysis. Recruitment started in October 2025 and is planned to continue until February 2027, with data collection until March 2027. Results are due for submission in October 2027. This protocol was developed in accordance with the SPIRIT (Standard Protocol Items: Recommendations for Interventional Trials) guidelines (S1 Checklist) [6]. The study was registered on 23^rd^ June 2025 (ISRCTN77648017) [7]. The current version of the study protocol is v2.0, 4^th^ December 2025 (S1 Protocol).

### Study setting and participants

The trial aims to recruit 494 participants from 29 NHS Trusts, including specialist children’s hospitals, tertiary units that treat children, and district general hospitals. Recruiting sites will be representative of the range of indices of deprivation [8]. Participants will primarily be recruited in emergency departments (ED), and in orthopaedic fracture clinics.

To be eligible for ToTs, patients must be aged from 9 months up to their 4th birthday at time of initial presentation to hospital, with a clinically suspected or radiologically confirmed toddler’s fracture of the tibia as determined by standard clinical practice at the recruiting site. They must not meet any of the following exclusion criteria: suspected non-accidental injury; associated displaced fibula fracture; comminuted/complex fracture patterns of the tibia; physeal injuries of the tibia; multiple fractures; metabolic bone disease; congenital anomalies involving the lower limb and foot (limb deficiencies); or previously participated in the ToTs study. Patients can take part in the study if they have received temporary immobilisation, as long as this can be removed within 3 days of initial presentation. Co-enrolment in other interventional studies is not permitted.

### Participant recruitment

Participating sites will make effort to recruit patients in person at their first ED attendance. Patients may also be identified at in person or virtual orthopaedic outpatient services (fracture clinics) or may be identified after discharge from hospital and then contacted via phone/videocall. Appropriately trained site staff will discuss the study with the patient’s parent/guardian and provide access to the information materials. This includes a short recruitment video animation, and an information sheet. Translations will be available in six languages other than English, which were chosen based the most common languages reported by sites in their expressions of interest, as well as consideration of the 2021 England & Wales Census.

Eligibility will be confirmed and informed consent taken from a parent/guardian with parental responsibility, by a trained and delegated clinician. Written consent will be taken place as soon as possible after patient identification and can be taken in person or remotely via phone/videocall and an online consent platform. Due to the age of participants, assent will not be obtained.

Prior to randomisation, baseline data will be collected, including patient’s postcode, contact details, ethnicity and sex. Parents/guardians will complete the primary outcome as a baseline measure.

### Randomisation and blinding

Randomisation will take place as soon as possible after initial presentation at the ED or fracture clinic, and no later than 3 days after initial presentation. Once eligibility is confirmed, and parent/guardian consent and baseline assessments are obtained, participants will be randomised 1:1, using an online system provided by the University of Sheffield Clinical Trials Research Unit (CTRU), to no-immobilisation or immobilisation, using minimisation with a random element and the following factors ensuring baseline balance: site, age (<=2 years versus >2 years) and radiologically confirmed fracture (yes versus no). The randomisation system will be set up by the study statistician. Randomisation will be done by site staff.

Blinding of participants and their parents/guardians, or those delivering the intervention, will not be possible. The trial statistician(s) will remain blinded at least until the statistical analysis plan has been signed off and approved by the oversight committees.

### Intervention and comparator

#### Immobilisation.

Participants will receive an above or below knee cast, or control action motion boot as per local standard practice. These should be applied in the ED, plaster room or fracture clinic by appropriately trained staff. If a cast is applied, the type used will depend on local policy and preference of the treating clinician (Plaster of Paris backslab or full cast, a synthetic soft cast, or a synthetic full cast). Casts will include an underlayer of wool with optional use of stockinette and adhesive felt for pressure areas. A temporary backslab may be used initially.

Treatment should be given as soon as possible after randomisation, and by the end of the third day after initial presentation. Immobilisation should continue for a minimum of 7 days after fitting, though sites will be advised that immobilisation should ideally remain in place for at least 2 weeks after it is given. Where a temporary backslab has been applied, change to a definitive immobilisation will be documented. On occasion, as part of standard care, a change of definitive immobilisation may be required if for example a cast becomes soft or damaged, or if complications arise. All such changes will be collected.

Participants will be followed up in ED/ fracture clinics as per the local site usual treatment pathways. Site standard protocols will determine the information given to parents regarding duration of wear, removal of immobilisation, care advice sheets, and analgesia.

Removal dates of immobilisation will be collected either from medical records or from questionnaires sent to parents/guardians, to allow for assessment of the fidelity of the intervention.

Participants will be considered ‘cross-over’ if they do not receive any immobilisation by day 3 after initial presentation, or if they remove their immobilisation within 7 days of fitting.

#### No-immobilisation.

If participants randomised to ‘no-immobilisation’ have received immobilisation prior to randomisation, this must be removed by the end of the third day after initial presentation to the ED, and as soon as possible after randomisation. Participants should not receive any immobilisation for at least 7 days post-randomisation, but ideally not at all within the trial period.

Parents/guardians can be offered a soft bandage for their child at the discretion of the site/clinician, but they do not need to accept it. Site standard protocols will determine the written information provided, and advice on analgesic use. Participants will be followed up as per usual treatment pathways.

Participants will be considered ‘cross-over’ if they do not remove any immobilisation fitted prior to randomisation by day 3 after initial presentation, or if they receive any new immobilisation after randomisation and before the 8^th^ day post randomisation.

#### Follow-up.

All parents/guardians will be given a questionnaire to complete at 3 days, 7 days, and 28 days after randomisation. Medical records will be reviewed by the site research team after 28 days. No other follow-up is required, but parents can contact the local hospital team during that 28-day period to discuss any concerns. Treatment adherence will be calculated via the 28-day medical note review, or questionnaires.

#### Outcomes.

The primary outcome for ToTs is pain, measured at 7 days post randomisation by the FLACC (Face, Legs, Activity, Cry, Consolability) behavioural pain assessment scale [9]. The FLACC score is commonly used to assess post-operative pain, and has been used for parental assessment of pain in research, with good reliability compared to medical staff administration [10–12]. The revised FLACC (rFLACC) will be used for children with cognitive impairment: this adds descriptors specific to the pain assessment of the child to ensure reliable pain assessments, and is validated for parental administration [13,14]. The FLACC scale (or rFLACC) will be used in accordance with standard guidance. The score ranges from 0–10 with higher values indicating higher levels of pain.

**Fig 1:**
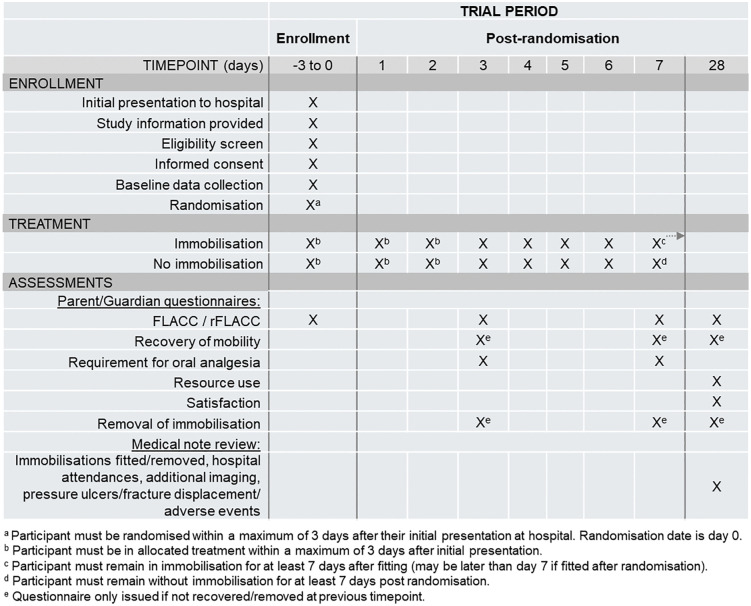
Schedule of enrolment, interventions, and assessments.

Secondary outcomes include the following after randomisation (see Fig 1 for specific timepoints and methods of collection):

- Pain (FLACC or rFLACC scale) at 3 days and 28 days.
- Recovery of mobility (time to weight bear).
- Requirement for and type of oral analgesia (up to day 7).
- Resource use.
- Satisfaction with allocated treatment.
- Planned and unplanned attendances to ED, plaster room, or fracture clinics.
- Use of plain radiograph imaging on the affected limb.
- Occurrence of and treatment for pressure ulcers, resulting from the use of immobilisation [15,16].
- Treatment for fracture displacement.

### Sample size

The sample size of 494 (247 per arm) was based on a non-inferiority margin of one point on the FLACC, an expected conservative standard deviation of 3.3 [17], with a one-sided alpha of 0.025, and 90% power, 15% loss to follow-up and a conservative estimate of correlation with baseline FLACC values of 0.3 [18].

### Participant retention

To promote retention, investigators will establish equipoise and discuss the importance of treatment adherence with parents/guardians before randomisation, but they are still able to change their child’s treatment if desired. If treatment is discontinued for clinical or personal reasons, participants will be encouraged to remain in follow-up for data collection.

Parents/guardians may withdraw consent for the study at any time, without providing a reason. If consent for active follow up (i.e., questionnaires) is withdrawn, sites will seek permission to continue accessing routinely collected clinical data. Any data collected up to the point of withdrawal will be retained for the final analysis, as specified during the consent process.

### Data collection and management

The parent/guardian questionnaires on day 3, 7 and 28 will collect the FLACC/rFLACC, recovery of mobility, analgesia use, resource use (asked at day 28 only), satisfaction (assessed by a Likert Scale and open free text question and asked at day 28 only), and treatment adherence. Questionnaires can be: sent automatically via text message as an online link to the study database; provided on paper along with a freepost envelope; or completed by phone call if necessary. The same parent/guardian will be encouraged to complete the questionnaires at all timepoints. Individual responses to questionnaires will not be monitored by hospital or CTRU staff, due to the short duration of the study, to avoid impacting on usual care.

Parents/guardians will be sent all questionnaires regardless of previous completion. Each timepoint includes two automated text reminders. As the primary outcome, the day 7 questionnaire may include a phone call reminder. Questionnaires will remain open until the end of the trial, with the final closing date set two weeks after the last participant’s 28-day follow-up.

The review of medical records after 28 days will collect information on immobilisation, attendances, x-rays, and complications, if it occurred within 28 days of randomisation, except for adverse events (AEs) which will be followed up to completion of the AE, or to the final participant completion.

Data management, protection, and archiving, will be provided by the University of Sheffield CTRU in accordance with their Standard Operating Procedures. Main study data will be captured and stored on CTRU’s in-house Prospect database), hosted on secure servers and in compliance with to NHS/University of Sheffield regulations and the UK Data Protection Act (2018) [19]. The system employs SSL/TLS encryption, encrypted password protection, and role-based privilege management. Quality control procedures will validate study data. Detailed project-specific procedures are detailed in a separate Data Management Plan. Data held by the CTRU and participating sites will be archived securely following completion in line with applicable regulations.

### Statistical analysis

The trial will be analysed and reported according to Consolidated Standards of Reporting Trials (CONSORT) guidelines for noninferiority designs [20]. Full details of all planned analyses and analysis populations will be collated in a pre-specified statistical analysis plan, which will be uploaded onto an online data repository when complete.

The primary endpoint (Day 7 FLACC score) will be analysed using a three-level mixed effects model with randomised treatment, follow-up time point (used as a categorical variable), randomised treatment by time point interaction, baseline FLACC score and minimisation variables (radiological diagnosis and age group) as fixed effects, and the post-randomisation FLACC scores at 3 days, day 7 (primary) and day 28 (level 1) nested within participants (level 2), nested within sites (level 3), with random intercepts at level 2 and 3. We will use restricted maximum likelihood estimation and assume an exchangeable covariance structure among the random effects.

The model will be used to obtain the marginal treatment effect (no-immobilisation vs. immobilisation) at 7 days post randomisation. Non-inferiority will be rejected if the upper limit of the 95% confidence interval exceeds the non-inferiority margin of one point in either the as-randomised or per-protocol population.

The as-randomised population will be based on the intention-to-treat principle, analysing all participants with available outcome data in line with their randomisation allocation, regardless of adherence to the protocol. The per-protocol population will be a subset of the as-randomised population, excluding participants who do not adhere to the protocol, as defined in the statistical analysis plan.

Treatment effects will also be presented for other timepoints.

The main analyses will use available data without imputation for missing data and assume that data are missing at random. Sensitivity analyses will assess the potential impact of missing data (including missing not at random scenarios), adherence to the randomised intervention (complier-average causal effects, if appropriate) and area-under-the-curve analyses to summarise cumulative pain over the follow-up. Consistency of treatment effects between important subgroups, including minimisation factors, will be explored via Forest plot, without the presentation of interaction tests.

Secondary endpoints will be analysed using comparable models for continuous and binary endpoints, as appropriate. Time to weight bear will be presented using summary statistics and compared between groups using a Cox proportional hazards model, adjusted for randomisation factors.

Adverse events and Serious Adverse Events (SAEs) will be presented descriptively. Index of Multiple Deprivation (IMD) deciles will be derived from postcodes and used to explore if treatment preferences and satisfaction differ across different IMD deciles.

### Health economic analysis

A primary economic evaluation will be undertaken from the NHS perspective using the within-trial 28-day timeframe. A secondary analysis will include a wider societal perspective. In the absence of a validated preference-based measure with an accepted set of preference weights for this age-group to generate quality adjusted life years [21], the primary outcome remains the most reliable way of measuring treatment benefit. Benefits in treatment will be calculated using area-under-the-curve of the FLACC scores at all timepoints. Resource use will be collected from all participants using a bespoke questionnaire at day 28, to include frequency of use of outpatient care, primary care, community care, social care and societal costs associated with medication, childcare and parent/guardians’ lost income. Unit costs will be taken from most recent standard sources [22,23], the British National Formulary [24] and NHS Supply Chain. The incremental cost-effectiveness ratio will be calculated by dividing the difference in mean costs of the treatments by the mean difference in the primary outcome. Probabilistic and deterministic sensitivity analyses will be undertaken to ascertain the robustness of the results. No long-term modelling will be conducted as it is expected that outcomes and costs will converge within the trial timeline [25].

### Patient and public involvement and engagement (PPIE)

Preliminary work for this study included an online survey of parents who previously underwent treatment (predominantly casted) at the Sheffield Children’s Hospital, Sheffield UK. Twelve of 14 responses said they would consider enrolling their child in a study randomising between immobilisation or no-immobilisation, assuming both were considered equal. Other data was gathered on expected difficulties in both arms, the length of follow-up, and how they would like to receive information. A group of parents from the Roma community were consulted on the outcome score and discussed how to ensure the trial is accessible to their community and others. Guidance from both of these groups was used to develop the study protocol and associated documents, and to choose the non-inferiority margin for the sample size collection; There was universal agreement that they would consider alternatives to immobilisation management for their toddler’s fractures only if this did not increase pain by more than one point in the FLACC score.

PPIE members helped to design and review patient facing documents and consider the delivery of participant information. During the study, PPIE co-applicants will be invited to Trial Management Group (TMG) meetings to provide input and feedback on how the study is running. Patient representatives will also sit on the TSC to provide their perspective on trial oversight. This is in addition to a wider PPIE group who will be consulted as and when PPIE input is important, as well as outreach to other charities to ensure accessibility of the study and of outputs. PPIE input into dissemination materials will be key to ensure they are fit for purpose, as well as consideration of where to disseminate trial results to ensure a wide audience is reached.

### Dissemination

The study results will be published in peer reviewed scientific journals and shared at clinical and academic conferences. Results will also be shared with participants who have requested to receive them and will be disseminated via social media in the form of a video animation.

### Oversight and monitoring

The study is sponsored and overseen by Sheffield Children’s Hospital and managed by the University of Sheffield. Study conduct, performance and safety will be overseen by three committees: a Trial Steering Committee (TSC), a Data Monitoring and Ethics Committee (DMEC), and a Trial Management Group (TMG) [26].

The DMEC will periodically review safety, progress and critical endpoint data reports. The TMG will receive reports from the TSC and DMEC to manage trial progress, and will review data reports including time from presentation to randomisation, and use of temporary immobilisation before randomisation, per site. Where necessary this will be reported back to the DMEC and TSC.

Annual remote monitoring of sites (with on-site triggers) will monitor adherence to the protocol and International Conference on Harmonisation Good Clinical Practice (ICH-GCP) [27] requirements. CTRU staff will regularly review entered data for possible errors and missing data points. Sites will securely share consent forms with CTRU for verification, as per the parents/guardian consent agreement.

### Adverse events

Adverse Events and SAEs are defined as an event that occurs after the participant has provided written informed consent for trial entry and within 28 days after randomisation. All AEs which are related or possibly related to the fracture or immobilisation/no-immobilisation will be recorded on the database, including those that fulfil the criteria for being serious. Unrelated AEs should not be recorded, unless they are deemed as serious. Pain itself does not need reporting as an AE, unless it meets the definition of being serious. Pressure ulcers and fracture displacement will not be recorded as adverse events, even if serious, as these are recorded as secondary outcomes.

### Ethics

The ToTs Study was given a favourable ethical opinion from West of Scotland Research Ethics Committee 5 (25/WS/0066) on 5^th^ June 2025*.* The study will be conducted in accordance with the protocol and ICH-GCP. The study will be submitted to local participating hospital Trusts to confirm capacity and capability before any research activity takes place.

Participant/parent/guardian confidentiality will be always respected. As per the consent form, identifiable data, including names, addresses and dates of birth, will be shared with CTRU to allow for participant follow-up. Participants will be allocated a unique identification number. This will be recorded on all data collection forms to preserve pseudonymity (except where identifiable information is collected). Both CTRU and the study sponsor, Sheffield Children’s Hospital [28], hold responsibility for ensuring that the trial complies with legislation and GCP.

### Protocol amendments

Any further amendments to the protocol will be agreed with the funder, sponsor, TSC, DMEC and TMG as required, and submitted to the Health Research Authority and Research Ethics Committee for approval. Amendments will also be notified to all sites and collaborating parties to confirm ongoing Confirmation of Capacity and Capability considering the new information. Parents/guardians will be notified and reconsented if appropriate to the change.

## Discussion

The ToTs trial addresses a critical gap in high-level evidence regarding the optimal management of toddler’s fractures. If non-inferiority is established, the clinical implications are substantial. Avoiding immobilisation would eliminate cast- or boot-related adverse events, such as pressure ulcers, skin breakdown, and joint stiffness, as well as avoiding issues of potentially delayed recovery, inconvenience to families, and the NHS and societal costs involved. Conversely, if non-inferiority is not established, this would validate the more prevalent practice of immobilisation, proving that the stabilisation provided by a cast or boot is indeed necessary for early pain control. Either way, the results will help standardise clinical guidelines.

A major strength of this trial is its pragmatic, multicentre, randomised controlled design. The findings will possess high external validity and generalisability across different demographics, and the incorporation of comprehensive PPIE ensured that the design, outcomes, and the non-inferiority margin reflect the priorities and acceptable thresholds of families. A key limitation is that blinding participants and their parents is impossible, which introduces a potential risk of bias in parent-reported outcomes. In addition, the trial relies on parental adherence to the randomisation allocation and follow-up schedule. While sensitivity analyses are planned to mitigate this, high crossover rates could still dilute the observed treatment effects.

By balancing pain management against the risks, costs, and inconveniences of immobilisation, this study will provide evidence on the optimal management of to these common childhood injuries.

## Supporting information

S1 Study Group The ToTs Study Group. (DOCX)

S1 Checklist SPIRIT Checklist. (DOCX)

S1 Protocol ToTs Study Protocol V2.0 04.12.2025. (PDF)
