# Supplementary material for: Treatment of Toddler’s Fractures: Study protocol for a multicentre non-inferiority RCT of no-immobilisation or immobilisation
Source: PLoS One. 2026 Aug 3;21(8):e0354801. doi: 10.1371/journal.pone.0354801 (PMC13432103; doi:10.1371/journal.pone.0354801)
Supplement: S1 Study Group — (DOCX) [file pone.0354801.s001.docx]

**The ToTs Study Group 15/05/2026**

In addition to those listed as authors on the manuscript ‘*Treatment of Toddler’s Fractures: study protocol for a multicentre non-inferiority RCT of no-immobilisation or immobilisation’*, the ToTs Study Group currently includes: Amanda Loban, Heather Dakin (*The University of Sheffield*), Rebecca Wetherill (*Sheffield Children’s Hospital*), Heather Jarvis (*Cardiff and Vale University Health Board*), Katie Hemmings-Trigg, Katherine Garner, Kelly Jones (*Northern Care Alliance NHS Foundation Trust*), Akshay Patel, Jessica Delaney, Laura Lee, Edward Snelson (*Norfolk and Norwich University Hospital*), Sarah Siner (*Alder Hey Children's Hospital Trust*), Marco Serafim (*Kingston and Richmond NHS Foundation Trust*), Aishwarya Prakash (*West Hertfordshire Teaching Hospitals NHS Trust),* Madelleine Barnett (*University Hospital Southampton NHS Foundation Trust*).
