# Supplementary material for: Treatment of Toddler’s Fractures: Study protocol for a multicentre non-inferiority RCT of no-immobilisation or immobilisation
Source: PLoS One. 2026 Aug 3;21(8):e0354801. doi: 10.1371/journal.pone.0354801 (PMC13432103; doi:10.1371/journal.pone.0354801)
Supplement: S1 Protocol — (PDF) [file pone.0354801.s003.pdf]

Treatment **o**f **T**oddler's fracture**s**: A multicentre non-inferiority RCT of observation or immobilisation

ToTs RCT

RESEARCH PROTOCOL
(Version 2.0) 4th December 2025

**IRAS Number:**	**353019**
**REC Reference:**	**25/WS/0066**
**Sponsor Reference:**	**SCH-2956**
**Funding Reference:**	**NIHR165783**
**ISRCTN:**	**ISRCTN77648017**
**Authorised by:**	**Mr Nicolas Nicolaou**

Sheffield Clinical Trials Research Unit (CTRU)

ToTs Study Protocol

Treatment of Toddler's Fractures: Observation Or Immobilisation

This document describes a clinical trial and provides information about procedures for entering participants. The protocol is not intended for use as a guide to the treatment of other patients. Amendments may be necessary; these will be circulated to known participants in the trial

Contents

Abbreviations **5**
Definition of terms 5
1. General information **6**
1.1 Investigator details 6
1.2 Clinical Trial Research Unit (CTRU) 7
1.3 Sponsor Details 8
1.4 Role of the Funder 8
1.5 Protocol amendments 8
1.6 Trial Summary 9
2. Introduction **12**
2.1 Background 12
2.2 Rationale for current study 13
3. Aims and objectives **15**
3.1 Aims 15
3.2 Objectives 15
4. Trial Design **15**
4.1 Blinding 16
5. Selection of participants **17**
5.1 Inclusion criteria 18
5.2 Exclusion criteria 18
5.3 Participant identification 18
5.4 Informed consent process 18
6. Randomisation and enrolment **19**
7. Trial treatment **20**
7.1 Patients randomised to ‘Immobilisation’ 20
7.2 Patients randomised to ‘No Immobilisation’ 21
7.3 Treatment adherence 21
8. Outcomes **22**
8.1 Primary outcome/endpoint 22
8.2 Secondary outcomes/endpoints 23
8.3 Internal pilot outcomes 24
9. Assessments and procedures **25**
9.1 Study assessments schedule 27
9.2 Unscheduled visits 28
9.3 Procedures for assessing efficacy 28
9.4 Procedure for assessing safety 28
9.5 Participant/parent/guardian withdrawals 28
9.6 Loss to contact 29
10. Safety Reporting **30**
10.1 Definitions 30
10.2 Recording and reporting 31
10.3 SAE notification procedure 31
10.4 CTRU responsibilities 32
10.5 SAE additional reporting 32
11. Statistics **32**
11.1 Sample size 32

11.2 Statistical Analysis 33
14. Trial supervision 34
14.1 Trial Steering Committee 35
14.2 Data Monitoring and Ethics Committee 35
14.3 Trial Management Group 35
15. Data handling and record keeping 36
15.1 Archiving 36
16. Data access and quality assurance 37
16.1 Site assessment 37
16.2 Risk assessment 38
16.3 Reporting serious breaches and non-compliances 38
16.4 On-site monitoring 38
16.5 Central monitoring 39
17. Publication 39
18. Finance 40
19. Ethics approval & regulatory compliance 40
20. Sponsor and site approval 41
21. Trial Organisation and Responsibilities 41
21.1 Principal Investigators 41
21.2 Sheffield Clinical Trials Research Unit (CTRU) 41
22. Patient & Public Involvement (PPIE) 42
23. Indemnity / Compensation / Insurance 43
24. References 44

Abbreviations

Definition of terms

AE	Adverse Event
CCC	Confirmation of Capacity and Capability
CI	Chief Investigator
CRF	Case Report Form
CTRU	Clinical Trials Research Unit
DMEC	Data Monitoring and Ethics Committee
ED	Emergency Department
GCP	Good Clinical Practice
GP	General Practitioner
HRA	Health Research Authority
HTA	Health Technology Assessment
ICH	International Conference on Harmonisation
ISF	Investigator Site File (This forms part of the TMF)
ISRCTN	International Standard Randomised Controlled Trials Number
NHS R&D	National Health Service Research & Development
NIHR	National Institute for Health and Care Research
PI	Principal Investigator
PPIE	Patient and Public Involvement and Engagement
RCT	Randomised Control Trial
REC	Research Ethics Committee
SAE	Serious Adverse Event
SCHARR	Sheffield Centre for Health and Related Research
SOP	Standard Operating Procedure
TMF	Trial Master File
TMG	Trial Management Group
TSC	Trial Steering Committee

1. General information

1.1 Investigator details

Chief Investigator:

Mr Nicolas Nicolaou
Department of Paediatric Orthopaedics and Spinal Surgery, Sheffield Children's Hospital National Health Service (NHS) Foundation Trust, Clarkson Street, Broomhall, Sheffield S10 2TH
Email: nicolas.nicolaou3@nhs.net
Tel: 07801 443331

Co-Lead Investigator:

Professor Shammi Ramlakhan
Department of Emergency Medicine, Sheffield Children's Hospital NHS Foundation Trust, Clarkson Street, Broomhall, Sheffield S10 2TH
Email: sramlakhan@nhs.net
Tel: 07960 547908

Co-applicants details:

Professor Steve Goodacre
Professor of Emergency Medicine, Department of Population Health, School of Medicine and Population Health, University of Sheffield

Dr Ines Rombach
Statistician, Sheffield Centre for Health and Related Research (SCHARR), University of Sheffield

Mr Richard Napier
Consultant Orthopaedic surgeon, Royal Belfast Hospital for Sick Children (RBHSC)

Dr Anju Keetharuth
Senior Health Economist, SCHARR, University of Sheffield

Ms Lizzie Swaby
Senior Study Manager/Research Fellow, Clinical Trials Research Unit (CTRU), SCHARR, University of Sheffield

Ms Fay Benskin
Parent and Patient Involvement and Engagement member

Miss Muniba Aslam
Patient and Public Involvement and Engagement (PPIE) Lead, Research and Innovation, Sheffield Children's Hospital NHS Foundation Trust

Mrs Sheryl Bennett
Parent and Patient Involvement and Engagement member

Name and address of an emergency contact in the event of the Principal Investigator (PI) /Chief Investigator (CI) becoming unavailable:

Professor Shammi Ramlakhan
Department of Emergency Medicine, Sheffield Children's Hospital NHS Foundation Trust, Clarkson Street, Broomhall, Sheffield S10 2TH
Email: sramlakhan@nhs.net
Tel: 0114 226 2331

1.2 Clinical Trial Research Unit (CTRU)

CTRU Oversights:
Robin Chatters
Email: r.chatters@sheffield.ac.uk
Tel: 0114 222 2969

Lizzie Swaby
Email: e.a.swaby@sheffield.ac.uk
Tel: 0114 222 4023

Statistician:
Ines Rombach
Email: i.rombach@sheffield.ac.uk
Tel: 0114 222 0840

Study Manager:
Katie Ridsdale
Email: k.ridsdale@sheffield.ac.uk
Tel: 0114 222 0746

Clinical Trials Research Unit (CTRU)

Sheffield Centre for Health and Related Research (SCHARR)

School of Medicine and Population Health

University of Sheffield

Innovation Centre c/o 30 Regent Street

Sheffield

S1 4DA

1.3 Sponsor Details

Ms Charlotte Heath
Sheffield Children's Hospital
Clarkson Street
Broomhall
Sheffield
S10 2TH
Email: charlotte.heath17@nhs.net

1.4 Role of the Funder

The funder has reviewed the research protocol but will have no role in data collection, analysis, data interpretation, report writing or in the decision to submit the report for publication. The funder has approved the selection of members for oversight committees.

1.5 Protocol amendments

Version number	Summary of changes
1.1	Inconsistencies corrected in flow diagram, study assessments schedule, and adverse event collection. Text adding regarding collection of demographic data.
1.2	Clarification about duration of immobilisation, and potential phone call reminders for questionnaires.
1.3	Addition of co-enrolment guidelines. Removal of collection of clinicial baseline treatment intent. Clarification of treatment adherence and immobilisation prior to randomisation. Clarification of pilot criteria. Addition of questionnaire completion by phone as option.

1.6 Trial Summary

Study title	Treatment of Toddler's fractures: A multicentre non-inferiority Randomised Controlled Trial of observation or immobilisation (ToTs)
Sponsor	Sheffield Children's Hospital
Funder	This study is funded by the National Institute for Health and Care Research (NIHR) Health Technology Assessment (HTA) Programme (NIHR165783).
ISRCTN	ISRCTN77648017
Project start date	1st January 2025
Project end date	31st October 2027
Aims	**Aims:**<br>• To discover whether no immobilisation is non-inferior to immobilisation in regard to pain at 7 days post randomisation, in children aged from 9 months up to and no later than their 4th birthday, who present with clinically suspected or diagnosed toddler's fractures.<br>• To explore whether no immobilisation has an impact on satisfaction with treatment, complications, and recovery time compared to immobilisation.
Trial design	1. A multicentre, prospective, parallel group, individually randomised (1:1), pragmatic, non-blinded controlled non-inferiority trial with 4 week follow up<br>2. Within-trial health economic analysis (NHS and a societal perspective).
Internal pilot/feasibility criteria	A 6-month internal pilot to assess feasibility of site set-up and recruitment, based on:<br>● Number of sites open and recruited 1st participant (target: 20)<br>● Number of participants recruited (target: 100)

	● Mean recruitment rate per site (target: average 1.63 per month)<br>● Availability of the primary outcome (number of recruited patients reaching and completing primary outcome measure) (target: 100%)<br>● Adherence to allocated treatment (100%)
Setting	NHS Emergency Departments (EDs) and Fracture clinics. Acute trusts that treat children.
Participants	**Inclusion Criteria:**<br>• Children aged from 9 months to their 4th birthday at time of initial presentation to hospital<br>• Clinically suspected or confirmed toddler's fracture of the tibia as determined by standard guidelines at the recruiting site.<br>**Exclusion criteria**<br>• Suspected non-accidental injury requiring further imaging or investigation<br>• Associated displaced fibula fracture<br>• Comminuted/complex fracture patterns of the tibia<br>• Physeal injuries of the tibia<br>• Multiple fractures<br>• Metabolic bone disease<br>• Congenital anomalies involving the lower limb and foot (limb deficiencies)<br>• Has previously participated in the ToTs Study
Intervention & control groups	Non-immobilisation<br>Immobilisation
Primary outcome(s)	Pain measured at 7 days post randomisation. Assessed by the FLACC (Face, Legs, Activity, Cry, Consolability) behavioural pain assessment scale. The revised FLACC will be used for children with cognitive impairment.
Secondary outcome(s)	Collected via medical note review at 28 days post randomisation:

	• Planned and unplanned attendances to ED, plaster room, or fracture clinics<br>• Use of plain radiograph imaging on the affected limb since randomisation<br>• Occurrence of and treatment for pressure ulcers, resulting from the use of immobilisation<br>• Occurrence of and secondary intervention for fracture displacement<br><br>Collected via parent/guardian questionnaires:<br>• Pain (via FLACC scale or Revised FLACC) at 3 days and 28 days post randomisation.<br>• Recovery of pre-injury mobility (time to weight bear) (asked at 3 days, 7 days and 28 days if mobility was not recovered by previous timepoint)<br>• Requirement for and type of oral analgesia up to day 7 (asked at 3 days and 7 days)<br>• Resource use and contact with General Practitioners since randomisation (asked at 28 days)<br>• Satisfaction with allocated treatment (asked at 28 days)<br>• Removal of immobilisation (asked at 3 days, 7 days and 28 days)
Duration of recruitment period and first enrolment date	Planned recruitment start: September 2025<br>Duration: 18 months, including 6 month pilot phase
Duration of follow-up	28 days
Target sample size	494 participants
Definition of end of trial	The end of trial is when the day 28 follow-up for the last participant is completed. Sites will be closed once data cleaning is completed and the ethics committee will be informed.

2. Introduction

2.1 Background

Toddler's fractures (non-displaced spiral fractures of the tibia) are common injuries sustained by children, often after a twisting injury to the leg following a stumble or fall. It involves the lower third of the tibia, and due to the thick lining of the bone (periosteum), the injuries heal quickly in a matter of weeks and are not thought to displace, thus avoiding long term problems. These injuries occur in up to 0.25% of children.[1] There are two broad groups of children: those with a clear fracture on x-ray; and those without. However, clinically both present after injury with an inability to bear weight through the leg as they normally would or bear any weight at all.

Treatment of these injuries is very variable in the United Kingdom (UK). Most clinicians will treat these injuries in a cast or boot that immobilises the limb to protect the injured leg, maintain alignment and promote comfort while it heals. Others opt to observe recovery from these injuries without any immobilisation, with pain relief provided as required. Research into current practice in a UK population is limited, but a retrospective review carried out in Scotland assessed 29 Toddler's fractures of which 12 were radiologically confirmed at diagnosis. Only one of the radiologically confirmed fractures was treated without a cast, in comparison to nearly half of those presumptively diagnosed.[2] A survey of Canadian ED clinicians found differences in treatment methods for these injuries suggesting variability in management is a universal finding.[3]

However, this treatment is not without issues, and there is a lack of good research into whether immobilisation is beneficial. A systematic review of the literature[1] identified 10 previous research studies (8 retrospective cohort studies and 2 randomised controlled trials) with a total of 963 participants aged 9-72 months (722 immobilised vs 241 non-immobilised). No significant difference was observed in fracture-related adverse outcomes between the groups, but 14.7% of immobilised children experienced non-fracture adverse events (pressure sores, fitting issues, breakage, pain, skin-related issues). There were no reported differences in discomfort or pain between the groups.

An older systematic review and meta-analysis of immobilisation vs no immobilisation identified four retrospective cohort studies comparing cast and no immobilisation for toddler's fractures.[4] All studies were small with a wide upper age range, although all included infants aged from 9 months. All studies included both radiologically confirmed

fractures and cases where the injury was not radiologically evident but clinically diagnosed. None of the studies commented on pain differences between treatment arms. A variety of immobilisation methods were used.

The more recent work of Fox et al conducted an RCT comparing immobilisation in a fibreglass long leg cast with no immobilisation.[5] This study identified higher complications in the immobilised group, but no difference in their selected outcomes. Participants were randomised between the ages of 9 months and 3 years, but of the 44 eligible subjects only 10 were randomised, the remaining 34 expressing a treatment preference. There was also racial bias as to which treatment preference was selected, with ‘non-white’ participants more likely to choose immobilisation. This is an important finding as it could be due to specific groups within the sample having a strong treatment preference. There were high satisfaction rates in the non-immobilised group. A non-validated modification of the Oxford Foot Ankle questionnaire was used as a patient reported outcome measure at presentation, 4 and 8 weeks. This may have missed symptoms of pain which are more likely to be experienced in both groups within the first 3 weeks. The study was powered to assess for differences in additional hospital visits and complications of treatment.

Overall, despite a low level of evidence, a risk of bias and lack of appropriate outcome measures, both immobilisation and non-immobilisation may be effective treatments. More evidence is therefore needed to assess whether these treatments differ in terms of participant pain, and satisfaction.

2.2 Rationale for current study

Treatment of toddler’s fractures via immobilisation of the leg can be associated with other complications. This includes pressure sores, skin breakdown, stiffness of the ankle and knee joint as well as pain from the cast rubbing. Use of immobilisation impacts on activities of daily life for children and their families. It also can lead to delayed recovery from the injury by limiting movement and causing temporary stiffness and weakness of the limb.

The potential advantages of a conservative approach (observation and no immobilisation) are ease of care of the child with a quicker recovery from the injury and no risk of complications from the cast or boot. Avoiding immobilisation allows free movement and prevents the need for frequent visits to check or remove the cast if

used. It prevents stiffness developing in joints that have been rigidly immobilised and potentially allows earlier return to normal activities once pain from the fracture has settled. Toddler's fractures are by definition stable injuries and tend not to displace if not immobilised. Furthermore, in cases where a toddler's fracture is the presumptive diagnosis but a bone or joint infection or minor injury is eventually diagnosed, immobilisation may delay definitive diagnosis.

However, there are concerns that without immobilisation there is a risk of the broken tibia displacing, that more pain is felt during recovery, and a risk of worsening of the injury. The inability to participate in normal activities such as attendance at nursery may also be affected. These may all impact on the child's and carer's wellbeing. Immobilisation allows the limb to be held still, relieving discomfort from the fracture associated with joint movement and maintains the ability to bear weight while the fracture heals.

Despite toddler's fractures being common injuries, there is no universally accepted way to treat these fractures with great variability in management across the UK and the rest of the world due to a lack of well conducted trials. The use of a cast may be an unnecessary treatment that causes discomfort, delayed recovery and unnecessary cost to healthcare organisations. There is therefore a requirement for a large-scale, high-quality randomised controlled trial comparing immobilisation vs no immobilisation.

A recent example of a paediatric orthopaedic randomised and controlled equivalence trial, the FORCE trial,[6] compared immobilisation in a removable splint to a bandage for torus fractures of the radius. There are some parallels with toddler's fractures in that both injuries heal quickly without long term problems, but inferring treatments from this study is difficult for a few reasons. Firstly, the tibia is a weight bearing bone and causes functional issues as well as pain. Secondly, use of a bandage in the upper limb is straightforward, but in the lower limb these slip off very easily and their use is therefore not standard. FORCE does however show that the planned treatment arms will be suitable for the trial.

As part of the preliminary work for this study, parents who had a toddler who previously underwent treatment at Sheffield Children's Hospital were contacted to take part in an online survey. The majority of these toddlers were treated with a cast. The survey asked if they would consider a study to allocate their child to treatment with or without a cast assuming both are considered equal. Of 14 responses, 12 responded 'yes'.

Other information was gathered on expected difficulties in both arms, the length of follow-up, and how they would like to receive information. This information was used to develop the study protocol and associated documents.

The study will be conducted in accordance with the protocol and International Conference on Harmonisation Good Clinical Practice (ICH-GCP).

3. Aims and objectives

3.1 Aims

- To discover whether no immobilisation is non-inferior to immobilisation in regard to pain at 7 days post randomisation, in children aged from 9 months up to and no later than their 4th birthday, who present with clinically suspected or diagnosed toddler's fractures.
- To explore whether no immobilisation has an impact on satisfaction with treatment, complications, and recovery time compared to immobilisation.

3.2 Objectives

1. To determine whether no immobilisation is non-inferior to management by immobilisation in this population in relation to pain by undertaking a multicentre RCT.
2. To determine patients' and parent/guardians' experience, recovery and satisfaction with the two treatments.
3. To evaluate the relative cost-effectiveness of no immobilisation compared to immobilisation by undertaking a within-trial economic evaluation from both an NHS and a societal perspective.

4. Trial Design

A multicentre, prospective, parallel group, individually randomised (1:1), pragmatic, non-blinded controlled non-inferiority trial with 4 week follow up and within-trial health economic analysis.

The trial aims to recruit 494 participants and will be conducted in approximately 20 NHS Trusts, including specialist Children's Hospitals; Tertiary units that treat children and District General Hospitals. Participants will be primarily recruited in Emergency

Departments, and in Fracture clinics if required. Eligibility will be confirmed by the researcher and study information given. Parents/guardians will be given time to consider the interventions following receipt of information about the study. Written consent will be taken from parents/guardians after any questions have been addressed. Due to the young age of participants, assent will not be obtained. Participants whose parents/guardians have consented will be randomised to receive either immobilisation, or no immobilisation, then followed up for 28 days.

A 6-month internal pilot, with clear progression criteria to full trial (see section 8.3) will follow the recommendations of Avery et al, and assess the feasibility of site set-up and recruitment.[7]

The internal pilot trial will use data from all sites which are open to recruitment within the first 6 months after the first site is opened. To allow time for collation of site and participant recruitment, and primary outcome, the progression criteria will be assessed by the Trial Steering Committee at the end of the following month. Data from the internal pilot will be included in the final analysis. At the end of the internal pilot phase, if any amber criteria are met, a recovery plan detailing remedial actions will be agreed with the Trial Steering Committee and submitted to the funder.

4.1 Blinding

Blinding of participants, or their parents/guardians (who complete the primary outcome assessment), or those delivering the intervention is not possible. The trial statistician(s) will remain blinded at least until the statistical analysis plan has been signed off and approved by the oversight committees. They may be unblinded thereafter to prepare for the final analysis.

5. Selection of participants

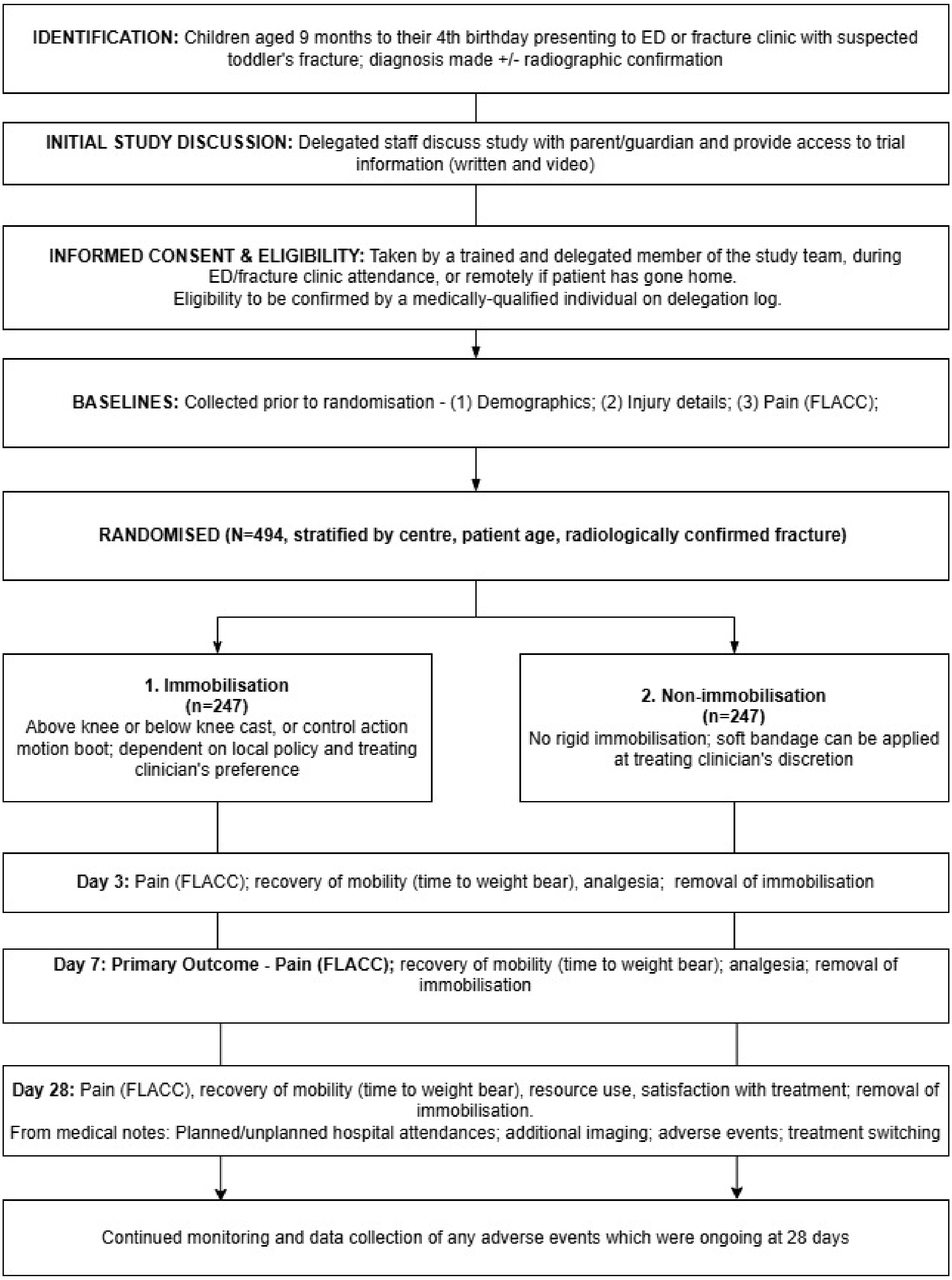

5.1 Inclusion criteria

Inclusion Criteria:

1. Children aged from 9 months to their 4th birthday at time of initial presentation to hospital
2. Clinically suspected or confirmed toddler's fracture of the tibia as determined by standard guidelines at the recruiting site.

5.2 Exclusion criteria

1. Suspected non-accidental injury requiring further imaging or investigation
2. Associated displaced fibula fracture
3. Comminuted/complex fracture patterns of the tibia
4. Physeal injuries of the tibia
5. Multiple fractures
6. Metabolic bone disease
7. Congenital anomalies involving the lower limb and foot (limb deficiencies)
8. Has previously participated in the ToTs Study

5.3 Participant identification

Sites should make every effort to recruit patients with suspected or confirmed toddler's fractures at their first attendance to emergency department (ED). Patients can also be identified at fracture clinics if necessary. Appropriately trained and delegated site staff will discuss the study with the parent/guardian, ideally in person, but with the option of phone/videocall if necessary, and provide access to the parent/guardian information materials. This includes a short recruitment animation, and a parent/guardian information sheet. Translations will be available in six languages other than English, which will be based on the 2021 Census, and information from recruiting sites on the common languages in their local authority areas.

5.4 Informed consent process

Eligibility will be confirmed by appropriately trained clinicians listed on the delegation log, and overall responsibility for eligibility assessment sits with the site PI. Interpreters will be available through standard NHS processes for the consent process, as required.

Informed consent will be taken from the parent/guardian (who has parental responsibility) by a trained and delegated member of the study team. This will ideally take place as soon as possible after identification, in person, during their ED attendance.

If patients are initially sent home from ED as part of routine care (before they are recruited to the study), but then the patient and parent/guardian present at a fracture clinic, consent may be obtained at the fracture clinic instead of at ED. Consent (and randomisation) can take place remotely if required, or requested by the parent/guardian, but the parent/guardian must be willing to return for their child's treatment if randomised to receive it.

Before consent, participants will have been provided with the patient information materials and will have had time to consider their potential participation. They will have the opportunity to ask any questions, before providing consent. They can take away the information to consider, if they request to do so. Parent/guardian consent must be obtained for the patient to be able to take part in the trial.

5.5 Co-enrolment guideliens

Co-enrolment in other interventional studies is not permitted during participation in ToTs. Consideration should be given to potential data collection burden associated with other concurrent studies, such as observational studies.

6. Randomisation and enrolment

Prior to randomisation, baseline data will be collected, including patients' post code, contact details, ethnicity and sex. Parents/guardians will complete the FLACC to provide a baseline measure.

Randomisation should take place as soon as possible after initial presentation at the ED or fracture clinic, and no later than three days after initial presentation. Ideally this will take place whilst the patient is still in the ED, but can be done in fracture clinic, or remotely, if necessary.

Once eligibility is confirmed, parent/guardian consent and baseline assessments are obtained, participants will be randomised 1:1, using an online system provided by the Sheffield CTRU, to no immobilisation or immobilisation, using minimisation with a random element and the following factors ensuring baseline balance: site, age (<=2 years vs. >2 years) and radiologically confirmed fracture (yes vs. no).

The initial participants will be allocated to their treatment using simple randomisation to seed the minimisation algorithm.

Patient details (Identification number, date of birth and stratification information) will be entered into the randomisation system and the treatment allocation will be returned. Randomisation will be done by site staff. Patients and their parents/guardians will be informed of their trial allocation in person (or via phone/video call if randomised remotely), and this will also be documented in the medical records. Their General Practitioner (GP) will also be informed of their participation in the trial, and their treatment allocation. Randomisation is Day 0 in regards to study follow-ups.

7. Trial treatment

7.1 Patients randomised to 'Immobilisation'

Participants should receive an above knee or below knee cast, or control action motion boot as per standard local practice. These should be applied in the ED, plaster room or fracture clinic by appropriately trained staff. If a cast, the type used will depend on local policy and preference of the treating clinician, as a Plaster of Paris backslab or full cast, a synthetic soft cast, or a synthetic full cast. Casts will include an underlayer of wool with optional use of stockinette and adhesive felt for pressure areas. Temporary backslabs may be used initially. The type of material used will be pragmatic and depend on the acute hospital standard care for cast application.

Treatment should be given as soon as possible after randomisation, and within three days of initial presentation (by the end of the third day) at the latest. Immobilisation should continue for at least 7 days after fitting, and ideally a minimum of 2 weeks after it is given. If participants were already in immobilisation prior to randomisation, the same immobilisation can remain on, if suitable.

Where a temporary backslab was applied, change to a definitive immobilisation will be documented. On occasion, as part of standard care, a change of definitive immobilisation may be required if for example a cast becomes soft or damaged, or if complications such as pressure sores arise. All changes will be collected.

Parents/guardians will be given written information about duration of wear, and removal of the immobilisation, as per site standard protocols. Sites will provide care advice sheets as per their standard protocol. Participants will be followed up in ED/ fracture clinics as per the local site normal treatment pathways. Advice as to analgesia will follow local routine care pathways.

Removal of immobilisation by parents/guardians will be recorded either in medical records or as part of questionnaires sent to parents/guardians, to allow for assessment of the fidelity of the intervention.

7.2 Patients randomised to 'No Immobilisation'

If participants have received immobilisation prior to randomisation, this must be removed within 3 days of initial presentation to the Emergency Department (by the end of the third day) at the latest, and as soon as possible after randomisation. Participants should not receive any immobilisation after randomisation for at least 7 days (until the end of the 7th day), but ideally not at all.

Participants can be offered a soft bandage at the discretion of the site/clinician. It is a personal choice if participants/parents/guardians would like to accept this or not. Parents/guardians may be given written information about duration of wear, application and removal of the bandage, as per site standard protocols.

Participants will be followed up as per usual treatment pathways at the local site. Advice on analgesic use will follow routine local care pathways.

7.3 Treatment adherence

Treatment change will be discouraged. Sites will discuss with parents/guardians the importance for the trial of remaining in their allocated treatment and will establish equipoise before the patient is randomised. However, if a parent/guardian still does wish to change their child's treatment after randomisation they can do so.

For participants randomised to the immobilisation arm, non-adherence is defined as follows:

- Removing the immobilisation prior to the 7th day after fitting (inclusive, i.e., up until the end of the 7th day),
- Not receiving any immobilisation by day 3 after initial presentation

For participants randomised to the non immobilisation arm, non-adherence is defined as follows:

- Receiving any new immobilisation before the 7th day after randomisation (inclusive, i.e. up until the end of the 7th day).
- Not removing any immobilisation fitted prior to randomisation by day 3 after initial presentation

Information on all immobilisation received during the will be recorded, including fitting/removal dates (from medical records/parent questionnaires), and the reason for change where available.

8. Outcomes

8.1 Primary outcome/endpoint

The primary outcome is pain measured at 7 days post randomisation. This will be assessed by the FLACC (Face, Legs, Activity, Cry, Consolability) behavioural pain assessment scale.[8] The revised FLACC will be used for children with cognitive impairment.[9]

Seven days was selected as our primary outcome based on our retrospective review of Toddler's fractures at Sheffield Children's Hospital, our PPIE work and survey of clinicians. This time point is identified as the most important for assessment as acute pain in the vast majority of children would have resolved and is the key point at which differences in treatment will be identified if taking baseline into consideration. PPIE consultation confirmed the use of this validated outcome measure with reporting via proxy would not overburden families.

8.2 Secondary outcomes/endpoints

Collected via medical note review by site staff at 28 days post randomisation:

- Planned and unplanned attendances to ED, plaster room, or fracture clinics
- Use of plain radiograph imaging on the affected limb since randomisation.
- Occurrence of and treatment for pressure ulcers, resulting from the use of immobilisation.[10,11]
- Occurrence of and treatment for fracture displacement.

Collected via parent/guardian questionnaires:

- Pain (via FLACC scale or Revised FLACC) at 3 days and 28 days post randomisation.
- Recovery of mobility (time to weight bear) (asked at 3 days, 7 days and 28 days post randomisation if mobility was not recovered by previous timepoint).
- Requirement for and type of oral analgesia up to day 7 (asked at 3 days and 7 days).
- Resource use and contact with GPs since randomisation (asked at 28 days post randomisation).
- Satisfaction with allocated treatment assessed by Likert Scale and open question (asked at 28 days post randomisation).
- Removal of immobilisation (asked at 3 days, 7 days and 28 days)

These timepoints are based on the minimal risk of long-term problems with these injuries and associated pain not persisting beyond 4 weeks due to healing.[12]

8.3 Internal pilot outcomes

A 6-month internal pilot to assess feasibility of site set-up and recruitment (Table 1).

Table 1: *Internal Pilot progression criteria*

	**Red: Consider ending study if at least TWO criteria are met.**	**Amber: Proceed with protocol amendments and remediation, as agreed with TSC**	**Green: Proceed**
No. of sites open and recruited 1st participant	<60% (n<12)	≥60% (n=12-19)	100% (n=20)
No. of participants recruited	<60% (n<60)	≥60% (n=60-99)	100% (n=100)
Mean recruitment rate per site	<50% (<0.8)	50-99% (0.8-1.62)	100% (1.63)
Proportion of participants completed primary outcome measure of those who reached it*	<85%	85-99%	100%
Adherence to allocated treatment**	<80%	80-99%	100%

**The number of participants for whom primary outcome data are expected allows for time for follow-up visits to be reached, as well as for reminders to be sent and data to be processed, including return of paper questionnaires. Therefore, this data will only be included for participants who were randomised 10 days prior (if via text) or 40 days prior (if via paper) to the date that the data is extracted for the analysis.*

***Adherence is defined as per section 7. This information will only be available when the 28 day medical note reviews are completed. This will therefore be calculated using data from participants for whom the 28-day medical note review has been completed at the time of assessing the pilot criteria.*

9. Assessments and procedures

The parent/guardians will be asked to complete the FLACC pain scale, either on paper or electronically, prior to randomisation.

During the trial follow-up, parents/guardians will be given a questionnaire to complete at 3 days, 7 days, and 28 days after randomisation. In most cases, this will be sent automatically via text, as an online link to the study database. If parents/guardians indicate that they would prefer to complete this on paper, the site staff will provide all questionnaires to the parent/guardian at point of randomisation, as well as a free-post return envelope to post these back to CTRU after all are completed. Questionnaires may also be collected over the phone if necessary, if questionnaires are not completed by the primary method. Parents/guardians will be encouraged to use the same parent/guardian completing the questionnaires at all timepoints. The importance of completing the questionnaires, in particular the FLACC questionnaire, will be discussed with the parent/guardian before they consent to take part. Up to two SMS reminders will be used to facilitate completion, plus a phone call reminder for the day 7 questionnaire, or paper questionnaires, if required. Questionnaires have been designed to be as simple as possible to decrease burden on the parent/guardian and increase response rate.

The FLACC pain scale will be asked at all timepoints. Other data will be gathered by bespoke questionnaires. At 3 days, and at 7 days post randomisation, parents/guardians will also be asked about analgesia use, recovery of mobility, and removal of immobilisation (if allocated to immobilisation). At 28 days post randomisation, parents/guardians will be asked about recovery of mobility (if not recovered by day 7), satisfaction with allocated treatment, and resource use.

Responses to questionnaires will not be monitored by hospital staff, or by CTRU staff, due to the short duration of study, and to avoid impacting usual care. Parents/guardians will be informed that these data will not be monitored, and will be told to contact their medical team if they have any concerns about their child. This will also be clear in the consent form and PIS.

The FLACC scale (or revised FLACC for children with cognitive impairment) will be used in accordance with the standard guidance. Each of the five categories of the FLACC scale (Face, Legs, Activity, Cry, Consolability) is scored from 0-2, which results

in a total score between zero and ten. The scores indicate: 0 = Relaxed and comfortable; 1-3 = Mild discomfort; 4-6 = Moderate pain; 7-10 = Severe discomfort/pain. The FLACC pain scale was developed to assess post-operative pain in children, and has been validated for the assessment of pain more generally, such as post trauma.[8] The revised FLACC (rFLACC) adds descriptors specific to the pain assessment of children with cognitive impairment, to ensure reliable pain assessments for this group of children.[9] We are using a combination of both scores, as appropriate to the characteristics of the toddler to ensure robust, unbiased data collection, and the best possible assessment of pain.

The FLACC has been used in a number of randomised controlled trials, and our PPIE group, clinical and methodological experts believe it to be the most appropriate primary outcome measure for this trial. Pain was chosen by our Patient and Public Involvement and Engagement (PPIE) group and clinicians as the most relevant outcome. The FLACC is one of two pain scores validated for this age group. This is validated for parental administration for children with cognitive impairment.[13] It is also commonly used to assess post surgery pain, and has been used for parental assessment of pain in research, which has shown good reliability compared to medical staff administration.[14–16] Our PPIE work showed that the format of the FLACC was easy for parents to understand and that measuring on 4 occasions was not a burden due to the ease of completion and the remote access that can be used if not aligning with standard clinical care.

Medical records will be reviewed by the site team after 28 days to identify any planned/unplanned healthcare attendances, use of plain radiograph imaging after randomisation, or complications within 28 days. Data collected from medical records is routine data and no clinician training is required.

9.1 Study assessments schedule

	Identification	Baseline	Treatment	3 days*	7 Days*	28 days*
**Enrolment**						
Screening form	S					
Eligibility form		S				
Informed consent form		S				
Demographics		S				
Injury details		S				
Randomisation (Day 0)		S (last)				
**Treatment**						
Treatment details			S			MR
**Primary outcome**						
FLACC (or revised FLACC)		Q		Q	Q (*primary*)	Q
**Secondary outcomes**						
Recovery of mobility				Q	Q **	Q **
Requirement for oral analgesia				Q	Q	
Resource use						Q
Parent/ guardian Satisfaction						Q
Attendances at ED, Plaster Room, or Fracture clinics						MR
X-rays on affected limb						MR
Pressure ulcers/ fracture displacement						MR
Removal of immobilisation				Q	Q **	Q ** / MR
**Safety**						
Adverse events						MR***

S = data collected in person/remotely by site staff
Q = data collected from parent/guardian via a self-completion questionnaire
MR = data collected from medical records
*post randomisation
**if not recovered/removed at previous timepoint
***Adverse events followed up until no longer ongoing

9.2 Unscheduled visits

Participants/parents/guardians may be seen at additional visits outside those contacts scheduled for the study, but these visits would be part of usual care. Any additional attendances will be recorded (from medical records) as part of the secondary outcomes.

9.3 Procedures for assessing efficacy

Efficacy will be assessed by comparing the mean FLACC score on day 7 (after randomisation) between the two groups.

9.4 Procedure for assessing safety

Adverse events and serious adverse events are discussed in Section 10. If the site research team have any concerns about a participant's wellbeing or safety during the course of the trial, this will be flagged to the patient's usual clinical team.

9.5 Participant/parent/guardian withdrawals

Due to the short follow-up period, and these timepoints aligning with usual care follow-up for these patients, withdrawal is expected to be minimal. Ability to complete outcomes remotely, with reminders, will also assist with this. We have kept the tasks required for participation as simple and infrequent as possible to ensure convenience without detracting from important outcomes. We have ensured that as much data is collectable as part of standard care without trial treatment visits and utilising online and text message-based data collection where possible.

Excessive participant/parent/guardian withdrawal from follow-up has a negative impact on a study. Centres will explain the importance of remaining on study follow-up to parents/guardians, and that changes to planned treatment need not imply withdrawal from the study. Parents/guardians may wish to stop their child's study treatment, or there may be a clinical need to stop study treatment (as per section 6.3). If this occurs, the parent/guardian should continue to complete their questionnaires, and sites should continue to collect information from medical records as per the study assessments schedule.

If parents/guardians do not wish to continue receiving questionnaires, their decision must be respected, and usual clinical care will continue. Parents/guardians may withdraw their consent for the study at any time, without providing a reason for this. If

this occurs, this will be documented on a study completion/ discontinuation form and the patient notes.

If parents/guardians withdraw their consent to continue receiving questionnaires, they will be given the option for their routinely collected data to be shared with the study team. This will allow the site to continue collecting information from their medical records as per the study assessments schedule, to inform the secondary outcomes.

If the parent/guardian explicitly states their wish for their child to not contribute further data to the study, this will be recorded on the study completion/discontinuation form, and no further data will be collected from the participant/parent/guardian for the study. Although the parent/guardian is not required to give a reason for discontinuing their study treatment, a reasonable effort will be made to establish this reason while fully respecting their rights.

Any data collected up to the point of the participant's withdrawal will be retained, and used in the final analysis, and this is made clear to the patient at the time of consent.

9.6 Loss to contact

Efforts will be made to contact parents/guardians, and questionnaires will be sent, for all follow-up timepoints, regardless of whether the parent/guardian has completed questionnaires at the previous time point. Two text message reminders will be sent per questionnaire time-point (at one and two days after the initial questionnaire is sent), plus a phone call reminder for the day 7 questionnaire, or paper questionnaires, if required. Questionnaires will be open for completion until the end of the trial. Date of completion will be collected and considered for the analysis. Questionnaires will close two weeks after the 28 day follow-up timepoint for the last recruited participant.

After the study closes, participants will be defined as lost to contact if no questionnaire data is available for one follow-up time point, and all subsequent stipulated follow-up time points.

10. Safety Reporting

ICH-GCP requires that both investigators and sponsors follow specific procedures when reporting adverse events in clinical studies. These procedures are described in this section.

10.1 Definitions

Term	Definition
Adverse Event (AE)	Any untoward medical occurrence in a study participant. *(refer to SOP PM004 Adverse Events and Serious Adverse Events for more details)*
Unexpected AE/SAE	An adverse event or serious adverse event which has not been pre-specified as expected.
Serious Adverse Event (SAE)	An AE which is serious, defined as any untoward medical occurrence or effect that :<br>● Results in death<br>● Is life-threatening*<br>● Requires hospitalisation or prolongation of existing inpatients' hospitalisation**<br>● Results in persistent or significant disability or incapacity<br>● Is a congenital anomaly/birth defect<br>● Is otherwise considered medically significant by the investigator***
Related AE/SAE	An AE or SAE which is related to a research procedure
Notable Event	An event of particular interest that does not necessarily meet the criteria for seriousness but requires expedited reporting as per the protocol.

*The term life-threatening in the definition of a serious event refers to an event in which the patient is at risk of death at the time of the event; it does not refer to an event that hypothetically might cause death if it were more severe, for example, a silent myocardial infarction.

**Hospitalisation is defined as an inpatient admission, regardless of length of stay, even if the hospitalisation is a precautionary measure for continued observation. Hospitalisations for a pre-existing condition, that has not worsened or for an elective procedure do not constitute an SAE.

***Other important medical events that may not result in death, be life-threatening, or require hospitalisation may be considered a serious adverse event/experience when, based upon appropriate medical judgement, they may jeopardise the patient and may require medical or surgical intervention to prevent one of the outcomes listed in this definition.

10.2 Recording and reporting

AEs and SAEs are defined as an event that occurs after the patient has provided written informed consent for trial entry and within 28 days after randomisation.

All AEs which are considered to be related or possibly related to the fracture or immobilisation/no immobilisation will be recorded on the database, including those that fulfil the criteria for being serious (see section 10.1). Unrelated AEs should not be recorded, unless they are deemed as serious. Sites are asked to enter all available information onto the study database as soon as possible after the site becomes aware of the event. Related AEs may be identified by site staff at any point during the study and should be recorded on the adverse event report form, within the participant CRF.

Pain in itself does not need reporting as an AE, unless it meets the definition of being serious.

The below AEs are secondary outcomes, and will be collected from medical records at day 28, and therefore recorded in a separate location on the database. AE report forms are not required for these. If these result in admission to hospital this does not require reporting as an SAE.

- Occurrence of and treatment for pressure ulcers resulting from the use of immobilisation
- Occurrence of and treatment for fracture displacement

SAEs (either related, or not related) will require more detailed information to be recorded on a PDF form. In such cases, the event must also be reported to the Sheffield CTRU within 24 hours of the site becoming aware of the event. The CTRU will coordinate ongoing monthly reporting to the Sponsor, or as soon as possible for an unexpected SAE. All SAEs will be reported, not just those related to the toddler fracture. The CI and/or co-CI will review all reported SAEs to ensure accuracy and consistency.

10.3 SAE notification procedure

CTRU should be notified of all SAEs (unless exempt), within 24 hours of the investigator becoming aware of the event.

The SAE form must be completed by the investigator or delegated member of the research team. All SAE forms must be sent by email to ctru-saes-group@sheffield.ac.uk. Receipt of the initial report should be confirmed within one working day. The site research team should contact the study team at CTRU if confirmation of receipt is not received within one working day.

Initial SAE reports must be followed by detailed reports when further information becomes available. Participants must be followed up until clinical recovery is complete, even if this is after the 28-day timeframe, and any laboratory or imaging results have returned to normal or baseline, or until the event has stabilized. Follow up information will be provided on an SAE report marked as such.

10.4 CTRU responsibilities

The Sponsor delegates CTRU responsibility for the reporting of SAEs to the regulatory authorities and the Research Ethics Committee (REC), as appropriate. CTRU will also keep all investigators informed of any safety issues that arise during the course of the study.

10.5 SAE additional reporting

The Data Monitoring and Ethics Committee (DMEC) and Trial Steering Committee (TSC) will also receive information on all AEs and SAEs, at a frequency agreed with each committee and documented in the appropriate charter/terms of reference.

11. Statistics

11.1 Sample size

The sample size of 494 (247 per arm) was based on a non-inferiority margin of one point on the FLACC, an expected conservative standard deviation of 3.3,[17] with a one-sided alpha of 0.025, and 90% power, 15% loss to follow-up and a conservative estimate of correlation with baseline FLACC values of 0.3.[18]

The non-inferiority margin was chosen based on face-to-face meetings with parents of children who had previously sustained a toddler's fracture. Pain levels in general and the FLACC scale in particular were discussed. Parents were asked to identify what difference in score they felt would reflect a difference in treatments for toddler's fractures. There was universal agreement that they would consider alternatives to

immobilisation management for their toddler's fractures only if this did not increase pain by more than one point in the FLACC score.

11.2 Statistical Analysis

The trial will be analysed and reported according to Consolidated Standards of Reporting Trials (CONSORT) guidelines for noninferiority designs.[19]

The primary endpoint, i.e. FLACC scores, will be analysed using a three-level mixed effects model with randomised treatment, follow-up time point (used as a categorical variable), randomised treatment by time point interaction, baseline FLACC score and minimisation variables as fixed effects, and the post-randomisation FLACC scores at 3 days, day 7 (primary) and day 28 (level 1) nested within participants (level 2), nested within sites (level 3), with random intercepts at level 2 and 3. We shall use restricted maximum likelihood estimation and assume an exchangeable correlation for the covariance structure between the random effects.

The model will be used to obtain the marginal treatment effect (non-immobilisation vs. immobilisation) at 7 days post randomisation. Non-inferiority will be rejected if the upper limit of the 95% confidence interval exceeds the non-inferiority margin of one point in either the as-randomised or per-protocol population. Treatment effects will also be presented for other timepoints.

Sensitivity analyses will assess the potential impact of missing data (including missing not at random scenarios), adherence to the randomised intervention (complier-average causal effects, if appropriate) and area-under-the-curve analyses to summarise cumulative pain over the follow-up. Consistency of treatment effects between important subgroups, including minimisation factors, will be explored.

Secondary endpoints will be analysed using comparable models for continuous and binary endpoints, as appropriate. Time to weight bear will be presented using summary statistics, and compared between groups using a Cox proportional hazards model, adjusted for randomisation factors.

(Serious) Adverse events (i.e. those not included in the secondary outcomes) will be presented descriptively.

Index of Multiple Deprivation (IMD) deciles will be derived from postcodes, and used to explore if treatment preferences and satisfaction differ across different IMD deciles.

Full details of all planned analyses and analysis populations will be collated in a pre-specified statistical analysis plan.

13. Economic evaluation

A primary economic evaluation will be undertaken from the NHS perspective using the within-trial 28-day timeframe. A secondary analysis will include a wider societal perspective. In the absence of a validated preference-based measure with an accepted set of preference weights for this age-group to generate quality adjusted life years,[20] the primary outcome remains the most reliable way of measuring treatment benefit. Benefits in treatment will be calculated using area-under-the-curve of the FLACC scores at day 3, day 7 and day 28. Resource use will be collected from all participants using a bespoke questionnaire at day 28, to include frequency of use of outpatient care, primary care, community care, social care and societal costs associated with medication, childcare and parent/guardians' lost income. Unit costs will be taken from most recent standard sources,[21] British National Formulary and NHS Supply Chain. To assess cost-effectiveness of the intervention, the incremental cost-effectiveness ratio (ICER) will be calculated by dividing the difference in mean costs of the treatments by the mean difference in the primary outcome. Probabilistic and deterministic sensitivity analyses will be undertaken to ascertain the robustness of the results. No long-term modelling will be conducted as it is expected that outcomes and costs will converge within the trial timeline.[22]

14. Trial supervision

The study will be led by the Chief Investigators working in coordination with the co-applicants and Sheffield CTRU. The Sponsor will be Sheffield Children's NHS Foundation Trust. Sheffield CTRU will take responsibility for project management and have set up a collaborator agreement for governance and safety reporting with the Sponsor. There is a dedicated study manager who is supervised by the CIs and senior staff in the CTRU, meeting regularly, and will liaise with the whole study team. There is also CTRU oversight for the delivery of all CTRU support including trial management, data management, quality assurance, randomisation, statistics, health economics,

analysis reporting and dissemination. Health Research Authority (HRA) approval will be sought prior to commencement of the trial at participating centres.

Three committees will govern study conduct, deliver the trial, monitor study performance and ensure its safety; TSC, DMEC and Trial Management Group (TMG). The committees will function in accordance with Sheffield CTRU Standard Operating Procedures (SOPs).

14.1 Trial Steering Committee

The TSC will consist of an independent chair, and at least three other members drawn from clinicians (with relevant clinical expertise), statisticians, health economists, and patient representatives. The role of the TSC is to provide supervision of the protocol, and statistical analysis plan, to provide advice on and monitor the study, to review information from other sources and consider recommendations from the DMEC. The TSC will meet at regular intervals, as defined in the TSC terms of reference. The TSC can prematurely close the trial, should this be recommended by the DMEC.

14.2 Data Monitoring and Ethics Committee

The DMEC will consist of at least three members, including an independent statistician, clinician and other independent member (i.e. clinician or trial methodologist). The DMEC will review reports provided by the CTRU to assess the progress of the study, the safety data and the critical endpoint data as required. The DMEC will meet at regular intervals, as defined by the DMEC charter, and meetings will comprise an open session to which members of the study team may attend, followed by a closed session with independent members only and to which unblinded data will be available. The DMEC may recommend the trial be stopped or modified on the basis of the data, in writing, to the chair of the TSC.

14.3 Trial Management Group

The TMG consists of the CIs, co-applicants and staff from CTRU, with site PIs and other site staff attending depending on need at each stage of the study. The CI will chair meetings to discuss the day-to-day running of the trial, including any implementation issues. The TMG will receive reports from the TSC and DMEC to manage trial progress. The study team will take reports to the TMG on time from presentation to randomisation, and use of temporary immobilisation before randomisation, per site, to assess if changes are required to recruitment pathways. Where necessary this will be reported back to the DMEC and TSC.

15. Data handling and record keeping

Participant/parent/guardian confidentiality will be respected at all times during the study. Data will be collected and handled in line with CTRU SOPs and in accordance with NHS Trust policies at Sheffield Children's Hospital NHS Foundation Trust and at each participating site. This will ensure systems are in place to protect confidentiality of participants/parents/guardians and the systems are secure.

Patients will be allocated a unique identification number that will be used to identify them throughout the trial. This will be recorded on all data collection forms to preserve pseudonymity (except where identifiable information is collected, such as on the contact details form, which will be kept separately).

All consent forms and questionnaires will be kept in a locked filing cabinet in a secured area. Sheffield CTRU may request consent forms to be sent from the research site to the CTRU via post or email as part of remote monitoring procedures. Parents/guardians will be asked to consent to this in the study consent form.

Data will be entered on to a secure study database, hosted on University of Sheffield servers and accessible over the internet, which adheres to data protection and NHS regulations. Identifiable data, including names, addresses and dates of birth, will be shared with Sheffield CTRU to allow for participant follow-up. Consent will be obtained from the patient for this to occur.

The investigator or delegate at each site will maintain comprehensive and accurate source documents to record all relevant study information regarding each participant, in all instances where the database does not form the source data.

15.1 Archiving

Data held by the CTRU will be stored in accordance with the archiving SOP (CTRU SOP PM012) for 10 years following completion. Archived documents will be logged on a register which will also record items retrieved, by named individuals, from the archive. Electronic data will be stored in an 'archive' area of the secure CTRU server for a minimum of 10 years. Archiving of the site files and participants' records at each participating centre will be the responsibility of the local R&D Department.

16. Data access and quality assurance

Direct access to source data/documents (including hospital records/notes, clinical charts, laboratory reports, pharmacy records and test reports) will be granted to authorised representatives from CTRU (study manager, research assistant, data managers, lead & Senior Research Nurses), the sponsor and host organisations to permit study related monitoring, audits and inspections. Select CTRU staff will have access to personal data including names, addresses, phone numbers and email addresses in order to undertake the questionnaire follow-up. In addition to this, access to the eCRF and questionnaire data will be required for study monitoring and audit purposes. A study monitoring plan will be devised in accordance with the Sheffield CTRU SOPs on Trial Monitoring (QU001).

The study database resides on Sheffield CTRU's in-house data management system. All data transmissions are encrypted using SSL/TLS, and access to the system is controlled by usernames and encrypted passwords. A comprehensive privilege management feature can be used to ensure that users have access to only the minimum amount of data required to complete their tasks. This will be used to restrict access to personal identifiable data. The database will incorporate quality control procedures to validate the study data. Discrepancy reports will be generated to highlight missing and erroneous information.

Overall responsibility for ensuring that each participant/parent/guardian's information is kept confidential will lie with the study sponsor. All paper documents will be stored securely and kept in compliance with the Data Protection Act (2018). Data entered onto the study database will be stored on CTRU servers at the University of Sheffield on behalf of the sponsors. After the trial has been completed and the reports published, access to the data will be strictly controlled.

16.1 Site assessment

Throughout this protocol, the trial 'site' refers to the hospital or clinic at which trial-related activities are conducted. Participating sites must be able to comply with:

- Trial treatments, imaging, clinical care, follow up schedules and all requirements of the trial protocol
- Requirements of the UK Policy Framework for Health and Social Care Research
- Data collection requirements

All site staff, including research staff, must be appropriately qualified by education, training and experience to perform the trial related duties allocated to them, which must be recorded on the site delegation log. CVs for all staff must be kept up to date, and copies held in the Investigator Site File (ISF), and the Trial Master File (TMF).

Before each site is activated, capability to conduct the trial will be assessed and documented. The CTRU will arrange a site initiation visit with each site or carry this out remotely. Site staff will be trained in the day-to-day management of the trial and essential documentation required for the trial will be checked. Once all the required documentation is in order and site staff have been trained, CTRU will formally activate the site to start recruitment. Sites should not open to recruitment until CTRU have provided this confirmation of activation.

16.2 Risk assessment

A risk assessment has been performed by the CTRU, in accordance with Sheffield CTRU SOPs.

Central and/or on-site monitoring will be undertaken at a level appropriate to the detailed risk assessment and will be documented in the Site Monitoring Plan.

16.3 Reporting serious breaches and non-compliances

A “serious breach” is a breach of either: the conditions and principles of GCP in connection with the trial or; the protocol relating to the trial; which is likely to effect to a significant degree –

- the safety or physical or mental integrity of the participants of the trial; or
- the scientific value of the trial

The sponsor will be notified immediately of any case where the above definition may apply during the trial conduct phase. The sponsor of a clinical trial will notify the REC in writing within 7 days of becoming aware of a serious breach.

All serious breaches and protocol non-compliances should be reported to CTRU within 24 hours of site staff becoming aware.

16.4 On-site monitoring

On-site or remote monitoring will be performed according to the monitoring plan and in line with the Sheffield CTRU Site Monitoring SOP.

A site initiation visit will be performed or carried out remotely for each participating site before each site recruits their first participant. During this visit/remote contact, the Monitor will review with site staff the protocol, study requirements and their responsibilities to satisfy regulatory, ethical and Sponsor requirements.

Regular site monitoring visits will occur throughout the study as specified in the Site Monitoring Plan and additional visits will be undertaken where required. At these visits, the Monitor will review activity to verify that the:

1. Data are authentic, accurate and complete.
2. Safety and rights of the patient are being protected and
3. Study is conducted in accordance with the approved protocol and study agreements, GCP and all applicable regulatory requirements.

Accurate and reliable data collection will be assured by verification and cross-check of the eCRF against Investigator's records by the Study Monitor (source document verification) (see section 13 for further details on data collection). Study Monitor will contact and visit sites regularly to inspect Case Report Forms (CRFs) throughout the study, to verify adherence to the protocol and completeness, consistency and accuracy of the data being entered on the CRFs.

A close-out visit will be performed after the last medical note review of the last patient at each site. Further close-out activities may be carried out remotely after this time, up to database freeze.

16.5 Central monitoring

CTRU staff will review entered data for possible errors and missing data points. A central review of consent forms will also be completed, and sites will be requested to share consent forms with CTRU via an NHS.net account, or password locked folders. This will be made clear to the parent/guardian prior to their consent to the trial.

17. Publication

Results of the study will be disseminated through peer reviewed scientific journals and at clinical and academic conferences, as well as submission of a final report to the funder, which will be made available online.

Other dissemination will take place through social media (e.g. facebook, X, mumsnet) and organisations of the investigators, using a dissemination video animation produced in six languages, This will be developed with PPIE input. Participants will be offered a summary of the results, co-produced with PPIE representatives.

Details of the study will also be made available on the Sheffield CTRU website. Summaries of the research will be updated periodically to inform readers of ongoing progress. The results will be published on a freely accessible database within one year of completion of the trial.

Full details, including guidance on authorship, will be documented in a Publication and Dissemination Plan.

18. Finance

ToTs is funded by the National Institute for Health Research (NIHR) Health Technology Assessment (HTA) Programme (NIHR165783). The views expressed are those of the author(s) and not necessarily those of the NIHR or the Department of Health and Social Care.

19. Ethics approval & regulatory compliance

Before initiation of the study at the participating site, the protocol, informed consent forms and information materials to be given to the parents/guardians will be submitted to West of Scotland Research Ethics Committee 5. Any further amendments will be submitted and approved by the HRA and ethics committee.

The study will be submitted to local participating Trusts to confirm Capacity and Capability before any research activity takes place.

Any amendments, including protocol modifications will be notified to all sites and collaborating parties to confirm ongoing Confirmation of Capacity and Capability (CCC) in light of the new information. Parents/guardians will be notified and reconsented if appropriate to the change.

20. Sponsor and site approval

Before initiation of the study at participating sites, the protocol, informed consent forms, and information materials to be given to the parents/guardians will require sponsor approval.

A site agreement between the Sponsor, participating sites and Sheffield CTRU outlines responsibilities of all parties and is to be signed prior to commencement of recruitment at sites.

Recruitment of study participants will not commence at a site until a letter of CCC has been issued.

21. Trial Organisation and Responsibilities

21.1 Principal Investigators

Each site will have a local Principal Investigator (PI) who will be delegated responsibility for the conduct of research at their centre and must sign a declaration to acknowledge these responsibilities. The local PI should ensure that all relevant staff involved are well informed about the trial and trained in study procedures, including obtaining informed consent and conduct of the trial according to GCP. The local PI will liaise with the Trial Manager on logistic and administrative matters with the trial.

21.2 Sheffield Clinical Trials Research Unit (CTRU)

The Sheffield CTRU at Sheffield University will provide set-up and monitoring of the trial conduct to CTRU SOPs and the GCP conditions and principles as detailed in the UK Policy Framework for Health and Social Care Research 2017. CTRU responsibilities include randomisation design and service, database development and provision, protocol development, CRF design, trial design, source data verification, monitoring schedule and statistical analysis for the trial. In addition, the CTRU will support the main REC, HRA and site-specific submissions, clinical set-up, on-going management including training, monitoring reports and promotion of the trial.

The CTRU Study Manager will be responsible for supplying investigator site files to each collaborating centre after relevant ethics committee approval and local R&D CCC has been obtained. The CTRU will be responsible for the day-to-day running of the

trial including trial administration, database administrative functions, data management, safety reporting and all statistical analyses. The CTRU will develop the site monitoring plan and data management plan and will assist the CI to resolve any local problems that may be encountered during the trial including any issues of noncompliance.

22. Patient & Public Involvement (PPIE)

PPIE contributors who were involved in the design of this study were also keen to be involved in future PPIE work to review patient information sheets, questionnaires and other patient facing materials. We plan to involve PPIE representatives during the following phases:

Set-up: We will work with the PPIE members to ensure the delivery of participant information, and questionnaires is optimal. PPIE contributors will review patient facing materials such as videos, information sheets, GP letters, as well as text reminders for questionnaires to ensure they are clear and provide the correct level of detail in a suitable format. PPIE input will also be crucial in the design of the study logo and poster.

Recruitment: PPIE will be involved to discuss the method by which potential participants and their parents/guardians are approached, taking into account any specific considerations important to parents/guardians.

During the study: The PPIE co-applicants will be invited to TMG meetings to provide their input on how the study is running, and feedback on recommendations from recruitment monitoring that will be triggered if recruiting centres struggle to consent eligible patients. Patient representatives will also sit on the TSC to provide their perspective in the oversight of the trial. This is in addition to a wider PPIE group who will be consulted as and when PPIE input is important.

Training: The PPIE Lead will provide training and support for PPIE co-applicants and group members. The Study Manager will be available to support PPIE representatives before, during and after TMG and TSC meetings, to ensure understanding, and answer any questions or provide clarification. A list of common research acronyms will also be provided to PPIE committee members to assist where these are not always explained in meetings.

Dissemination and impact: PPIE input into dissemination materials will be key to ensure they are fit for purpose, as well as consideration of where to disseminate trial results to ensure a wide audience is reached.

23. Indemnity / Compensation / Insurance

The University of Sheffield has in place clinical trials insurance against liabilities for which it may be legally liable and this cover includes any such liabilities arising out of this clinical study.

Standard NHS indemnity operates in respect of the clinical treatment that is provided.

24. References

1. Ramlakhan S. Management of Toddler's fracture: A systematic review with Meta-analysis. Unpublished.

2. Sapru K, Cooper JG. Management of the Toddler's fracture with and without initial radiological evidence: European Journal of Emergency Medicine. 2014 Dec;21(6):451–4.

3. Chen SN, Holstine JB, Samora JB. Reducing Rigid Immobilization for Toddler's Fractures: A Quality Improvement Initiative. Pediatric Quality & Safety. 2024 Mar;9(2):e722.

4. Boutin A, Misir A, Boutis K. Management of Toddler's Fracture: A Systematic Review With Meta-Analysis. Pediatr Emer Care. 2022 Feb;38(2):49–57.

5. Fox J, Enriquez B, Bompadre V, Carlin K, Dales M. Observation Versus Cast Treatment of Toddler's Fractures. Journal of Pediatric Orthopaedics. 2022 May;42(5):e480–5.

6. Perry DC, Achten J, Knight R, Appelbe D, Dutton SJ, Dritsaki M, et al. Immobilisation of torus fractures of the wrist in children (FORCE): a randomised controlled equivalence trial in the UK. The Lancet. 2022 Jul;400(10345):39–47.

7. Avery KNL, Williamson PR, Gamble C, O'Connell Francischetto E, Metcalfe C, Davidson P, et al. Informing efficient randomised controlled trials: exploration of challenges in developing progression criteria for internal pilot studies. BMJ Open. 2017 Feb;7(2):e013537.

8. Merkel S, Voepel-Lewis T, Shayevitz J, Malviya S. The FLACC: A Behavioral Scale for Scoring Postoperative Pain in Young Children. Pediatric nursing. 1996 Nov 30;23:293–7.

9. Malviya S, Voepel-Lewis T, Burke C, Merkel S, Tait AR. The revised FLACC observational pain tool: improved reliability and validity for pain assessment in children with cognitive impairment. Pediatric Anesthesia. 2006 Mar;16(3):258–65.

10. Coleman S, Smith IL, Nixon J, Wilson L, Brown S. Pressure ulcer and wounds reporting in NHS hospitals in England part 2: Survey of monitoring systems. Journal of Tissue Viability. 2016 Feb;25(1):16–25.

11. Smith IL, Nixon J, Brown S, Wilson L, Coleman S. Pressure ulcer and wounds reporting in NHS hospitals in England part 1: Audit of monitoring systems. Journal of Tissue Viability. 2016 Feb;25(1):3–15.

12. Adamich JS, Camp MW. Do toddler's fractures of the tibia require evaluation and management by an orthopaedic surgeon routinely? European Journal of Emergency Medicine. 2018 Dec;25(6):423–8.

13. Voepel-Lewis T, Malviya S, Tait AR. Validity of Parent Ratings as Proxy Measures of Pain in Children with Cognitive Impairment. Pain Management Nursing. 2005 Dec;6(4):168–74.

14. Uitti JM, Salanterä S, Laine MK, Tähtinen PA, Ruohola A. Adaptation of pain scales for parent observation: are pain scales and symptoms useful in detecting pain of young children with the suspicion of acute otitis media? BMC Pediatr. 2018 Dec;18(1):392.

15. McGrath M, Kim J, Farrokhyar F, Braga LH. Randomized Controlled Trial of Scrotal versus Inguinal Orchidopexy on Postoperative Pain. Journal of Urology. 2021 Mar;205(3):895–901.

16. Rybojad B, Sieniawski D, Rybojad P, Samardakiewicz M, Aftyka A. Pain Evaluation in the Paediatric Emergency Department: Differences in Ratings by Patients, Parents and Nurses. IJERPH. 2022 Feb 21;19(4):2489.

17. Crellin DJ, Harrison D, Santamaria N, Babl FE. Comparison of the Psychometric Properties of the FLACC Scale, the MBPS and the Observer Applied Visual Analogue Scale Used to Assess Procedural Pain. JPR. 2021 Mar;Volume 14:881–92.

18. Sample Size Software | Power Analysis Software | PASS | NCSS.com [Internet]. [cited 2025 Mar 3]. Available from: https://www.ncss.com/software/pass/

19. Piaggio G, Elbourne DR, Pocock SJ, Evans SJW, Altman DG, Consort Group FT. Reporting of Noninferiority and Equivalence Randomized Trials: Extension of the CONSORT 2010 Statement. JAMA. 2012 Dec 26;308(24):2594.

20. Rowen D, Rivero-Arias O, Devlin N, Ratcliffe J. Review of Valuation Methods of Preference-Based Measures of Health for Economic Evaluation in Child and Adolescent Populations: Where are We Now and Where are We Going? PharmacoEconomics. 2020 Apr;38(4):325–40.

21. NHS England » 2021/22 National Cost Collection Data Publication [Internet]. 2023 [cited 2025 Mar 3]. Available from: https://www.england.nhs.uk/publication/2021-22-national-cost-collection-data-publication/

22. Adamich JS, Camp MW. Do toddler's fractures of the tibia require evaluation and management by an orthopaedic surgeon routinely? European Journal of Emergency Medicine. 2018 Dec;25(6):423–8.
